# Beyond the hospital infection control guidelines: a qualitative study using positive deviance to characterize gray areas and to achieve efficacy and clarity in the prevention of healthcare-associated infections

**DOI:** 10.1186/s13756-018-0418-x

**Published:** 2018-10-24

**Authors:** Anat Gesser-Edelsburg, Ricky Cohen, Adva Mir Halavi, Mina Zemach, Peter Vernon van Heerden, Sigal Sviri, Shmuel Benenson, Uriel Trahtemberg, Efrat Orenbuch-Harroch, Lior Lowenstein, Dan Shteinberg, Asher Salmon, Allon Moses

**Affiliations:** 1 School of Public Health, University of Haifa, 199 Aba Khoushy Ave., Mount Carmel, 3498838 Haifa, Israel; 2 The Health and Risk Communication Research Center, University of Haifa, 199 Aba Khoushy Ave., Mount Carmel, 3498838 Haifa, Israel; 3 Midgam Consulting & Research Ltd., 7 Metsada St, 5126112 Bnei Brak, Israel; 4 Hadassah University Medical Center. Ein Kerem, P.O. Box 12000, 9112001 Jerusalem, Israel; 5 Rambam Health Care Campus, P.O. Box 9602, 3109601 Haifa, Israel; 6 Bnai Zion Medical Center, 47 Golomb St, P.O.B. 4940, 3104802 Haifa, Israel

**Keywords:** Infection control guidelines, Positive deviance approach, Gray areas, Efficacy and clarity, Qualitative study

## Abstract

**Background:**

The literature is replete with attempts to design and promote customized guidelines to reduce infections during the care continuum. Paradoxically, these efforts sometimes result in gray areas where many staff members are unaware of what is required of them, which then leads to confusion, frustration, and uncertainty.

We coined the phrase “gray areas” in this context to encompass the variety of situations on the care continuum that are not addressed in the accepted guidelines, and where staff members are unsure of how to proceed.

The purpose of the present study was to characterize the gray areas that were reported by staff and to identify the practices of Positive Deviance (PD) individuals. We define to PD individuals as people who independently develop creative solutions to solve problems not identified by the majority in their community.

**Methods:**

A qualitative constructivist research methodology was used that included personal interviews, observations and video recordings of identified PD practices to enhance infection control. The study was conducted January through March 2018, in two Intensive Care Units (ICU) units at Hadassah Hospital, Jerusalem, Israel. Personal interviews were conducted with 82 staff members from the General ICU (GICU) and Medical ICU (MICU).

**Results:**

The study confirmed that guidelines cannot cover all the different situations that arise during the care continuum and can paradoxically result in the increased spread of hospital infections. Our study found there are numerous individuals who independently develop and implement solutions for gray areas. The creative and practical solutions of PD individuals can address the barriers and difficulties on the care continuum that were encountered by the staff in their communities. For example, inserting a central venous line is a complex practice in the general guidelines, while the PDs provided clear situation-specific solutions not covered in the guidelines.

**Conclusions:**

The recommendations of the present study are to encourage hospital personnel to create their own solutions for various situations on the care continuum, and to disseminate them within their units to achieve a bottom up change, in lieu of investing in new or specific written guidelines.

**Electronic supplementary material:**

The online version of this article (10.1186/s13756-018-0418-x) contains supplementary material, which is available to authorized users.

## Background

Healthcare-associated infections (HAI) are one of the more complex problems in healthcare that no country or organization, despite multiple efforts, have managed to completely solve. The World Health Organization (WHO) claims that hundreds of millions of patients are affected by HAIs worldwide annually, which is not only a serious health issue, but also results in a financial drain on for the health system [1, 2].

Annually, approximately 4,544,100 infections and 37,000 fatalities are reported in the European Union (EU), and 2,000,000 infections and 100,000 fatalities in the United States (US), as a result of acquired infections transmitted through healthcare workers [3, 4]. A State Comptroller Report reported an estimated annual incidence of 40,000–100,000 HAI in Israel that resulted in 4000–6000 fatalities in 2012 [5].

HAI’s can be reduced through infection prevention and control (IPC) practices based on evidence-based guidelines that are practical and feasible [6]. Guidelines and practices have been developed to help hospital workers reduce HAI. During the 1970’s and 1980’s, the Center for Disease Control and Prevention (CDC) produced guidelines, including instructions for hand washing with non-antimicrobial soap. The guidelines were appropriate, but hospitals lacked implementation policies for medical personnel not in direct contact with patients; such as technicians, laundry workers and hospital orderlies [7]. The guidelines were modified to clearly delineate different situations when hand washing was mandated, and the use of alcohol-based antiseptics for preventing contamination before and after contact with patients [8]. In 2004, the Institute for Healthcare Improvement (IHI) initiated a “100,000 Lives” campaign with six strategies to reduce morbidity and mortality in hospitalized patients, which included three HAI prevention methods to reduce central-line infections, surgical site infections and ventilator-associated pneumonia [9].

In 2009, updated hand hygiene (HH) guidelines were developed by the WHO, highlighting a new method called “My Five Moments for Hand Hygiene.” The method includes systemwide changes, education and training, performance feedback, reminders in the workplace, and how to develop a safety climate. The vision was to develop guidelines that could be implemented at all income levels worldwide [10].

The latest WHO IPC guidelines, published in 2016, aim to improve practices with more effective and feasible guidelines, based on available resources, public health needs, and the local background. This includes the addition of water sanitation and hygiene (WASH), environmental and human factors, bed occupancy and staffing ratios, HH monitoring with feedback and the use of multi-modal strategies [11].

Very few studies in the literature have tried to analyze why, despite multiple efforts to make these guidelines operable, there remains a significant disparity between the guidelines and their implementation in the field. In one study Gurses et al. [12] tried to solve this problem and analyze its complexity. The research was conducted in 2006, in two separate teaching hospitals, in surgical ICUs. The study focused on four evidence-based guidelines: ventilator-associated pneumonia, central venous catheter-related bloodstream infections, surgical site infections, and catheter-associated urinary tract infections. Five subcategories of ambiguity relating to guideline discrepancies emerged from the study and included task, expectation, responsibility, method and exception ambiguity [12].

The gap between guidelines and maintaining them in the field has given rise to numerous intervention programs by public health workers. Despite the variety of interventions conducted for healthcare workers, the levels of compliance with HH still remain low at 50–60% [13–15]. A systematic review of intervention programs by Srigley et al. [16] concluded that interventions based on behavioral models were more successful in raising compliance with HH than interventions that only addressed knowledge and awareness.

According to Singhal [17] “the Positive Deviance (PD) approach is based on the premise that in every community there are certain individuals or groups whose uncommon behaviors and strategies enable them to find better solutions to problems than their peers, while facing worse challenges and having access to the same resources. However, these people are ordinarily invisible to others in the community.” The PD approach differs from common approaches to problem-solving, as it seeks to identify and streamline existing resources that are derived from the staff within the unit, rather than import external “best practices”. The approach focuses on the positive identification of solutions rather than problems. The PD approach identifies the behavioral practices of positively deviant individuals within the community and builds a social network to distribute and implement those practices over time [18, 19]. PD has been used to address the problem of hospital infections in the USA [20–23]. Global studies have shown that implementing the PD approach results in a significant and lasting improvement in staff compliance with guidelines for preventing infections, and a drop in the number of HAI’s at healthcare centers. For example, a PD intervention was implemented across Veterans Affairs hospitals in the USA. The rate of healthcare-associated MRSA infections in ICUs before intervention was 1.64 infections per 1000 patient-days on October 2007, which was reduced to 0.62 infections per 1000 patient-days post-intervention on June 2010, a decrease of 62% (*P* < 0.001). The rate of HAI Methicillin-resistant *Staphylococcus aureus* (MRSA) infections in non-ICUs fell from 0.47 per 1000 patient-days to 0.26 infections per 1000 patient-days post intervention, a decrease of 45% (P < 0.001) [18, 20, 24].

Most studies to date have focused on evaluating the effectiveness of intervention programs with healthcare workers rather than identifying the reasons for the existing gaps between the written guidelines and their implementation, as Gurses et al. [12] pointed out in his study, we defined as “gray areas”. “Gray areas” are the variety of situations on the care continuum that are not addressed by the accepted guidelines and where staff members were unsure how to proceed.

The purpose of the present study was to characterize the gray areas in the care continuum in ICUs where systematic guidelines are adhered to only partially by the staff, and where there are no practices of PD individuals that address these “gray areas” as reported by the staff.

## Methods

### Research design

A qualitative constructivist research method used personal interviews with staff members in different sectors, observations on the ground, and video recordings of identified positive behavioral practices to maintain hygiene. We also examined the gap between existing guidelines for hygiene maintenance and implementation in situ.

Sampling method and participant recruitment: The study was conducted January through March 2018, in two ICU’s at Hadassah Hospital, Jerusalem, Israel. We chose to focus on ICU’s where staff members are exposed to significant work stress, patient mortality, and feelings of professional frustration. These pressures increase the gap between the guidelines and their implementation [25–28]. Therefore, it is important to identify solutions and disseminate them in these units.

We used several sampling strategies during the study and interviewed over 90% of the staff in the GICU and MICU. In the first stage, intensive and heterogeneous sampling was used to include all representative sectors in the units (physicians, nurses, cleaning staff, etc.). In the second stage, we used snowball sampling [29] which is a method that identifies desired individuals (e.g. PDs) based on their colleagues’ recommendations during the first interview. The advantage of snowball sampling is that information, usually hard to unveil, can be identified, especially when the behavior or lifestyle of the individual is an exception to the norm. In the third stage, we interviewed people identified as PDs by staff or the research team.

### Study population

At Hadassah Hospital, a total of 82 participants were interviewed from the GICU and MICU: 47 nurses, 14 physicians, 5 nursing aides, 5 nursing students, 2 social workers, 2 physical therapists, 1 respiratory technician, 2 secretaries, 1 national service volunteer and 3 cleaning staff (Table 1).

**Table 1:** Interviewees: sociodemographic characteristics (*n* = 82)

Sociodemographic characteristics	Category	*n* (%)
Gender	Men	25 (30.5)
	Female	57 (69.2)
Age (years)	Mean (Max, Min, SD)	35.3 (67, 18, 11.3)
Ethnicity	Jewish	62 (75.6)
	Arab	20 (24.4)
Country of Origin	Israel	58 (74.4)
	Other	24 (29.3)
Tenure (years)	Mean (Max, Min, SD)	8.7 (40, 1, 8.6)
Position	Nurse	47 (57.3)
	Physician	14 (17.1)
	Nursing aide	5 (6.1)
	Nursing student	5 (6.1)
	Cleaning staff	3 (3.7)
	Other	8 (9.8)
Department	GICU	45 (54.9)
	MICU	37 (45.1)

### Research tools

We triangulated the data obtained from different sources to bolster the study’s validity: face-to-face interviews, observations and video. We also strengthened validity after the snowball sampling by obtaining confirmation from the Infection Control Unit (two physicians and an infection-control nurse) regarding the PD practices found. Interviews and observations were conducted alternatively in different day shifts for periods of several hours and PD behaviors were documented in detail in a field notes. Social Network Maps were produced using Social Network Visualizer 2.3 [30].

#### Interviews

Before each interview, staff members received an explanation about the study and its goals and signed an informed consent form. The semi-structured interview protocol (Additional file 1: Table S2) included questions regarding difficulties in maintaining infection control guidelines, risk perceptions of infectious diseases, norms, and the hospital’s organizational culture. Interviewees were asked to name staff members they believed to be PD, defined as: persons who demonstrated positive deviant behaviors to maintain HH or who raised ideas for such practices. The interview protocol was based on Discovery & Action Dialogue (DAD) guidelines that are based on the PD approach [31]. The interviews elicited gray areas when we asked for situations lacking clear guidelines. Subsequently, interviewees were asked to identify staff who they thought had positive behavioral practices that addressed these “gray areas” effectively.

#### Observations

In the first stage, observations were made of all unit staff members’ infection control maintenance practices of concern on the care continuum, as well as attitudes towards procedures. In the second stage, we conducted focused observations only of PD individuals.

#### Video footage

Consenting staff members, identified in the interviews and observations as PD, were filmed performing the positive practices during their work. The use of this tool is based on Bandura’s theory of social learning, which says that most human behavior is influenced and learned by observing the behavior of others [32]. The videos were important for designing and developing activities to spread the PD solutions, to help community members learn and practice the positive behaviors.

The data was first gathered via interviews, transcribed, observed and the main barriers and PD practices identified through content analysis. The data was then further analyzed using content analysis to find sub-themes derived from the main gray area themes, and to classify practices that stood out during the PD interviews. Throughout the study the researchers who collected the data via interviews and observations, reflexively examined themselves so as not to be judgmental or critical and to only focus on discovering positive practices.

### The research processes

#### Stage I

Research documents were prepared, submitted and approved by the ethics committee of The Faculty of Social Welfare and Health Sciences at the University of Haifa (confirmation number 392/17) and by the Bnai Zion Medical Center Helsinki Committee (confirmation number 135–16-BNZ).

#### Stage II

A meeting was held with administration representatives from the Infection Control Unit, where the research goals, plan and PD approach were presented, and cooperation requested. After brainstorming and based on the hospitals’ needs, the General and Medical ICU units were selected for the research.

#### Stage III

One meeting was held with multi-sectorial representatives (physicians, nurses, nurses’ aides, orderlies and cleaning staff) in each unit, during which an explanation was provided about the research goals, framework and the PD approach.

#### Stage IV

The researchers entered the units with the unit head nurse and received a tour of the physical structure of the unit and introduction to shift staff. Subsequently they visited each unit twice a week, on different days, shifts and hours, during which general and direct observation was conducted of staff practices, and semi-structured protocol interviews were held. The interviews were recorded and transcribed by the researchers and all observations documented at the end of each observation. The researchers were trained to conduct interviews using the DAD method by the research supervisor, who is in an expert in qualitative studies.

## Results

### Classifying the PD’s

**Fig. 1:**
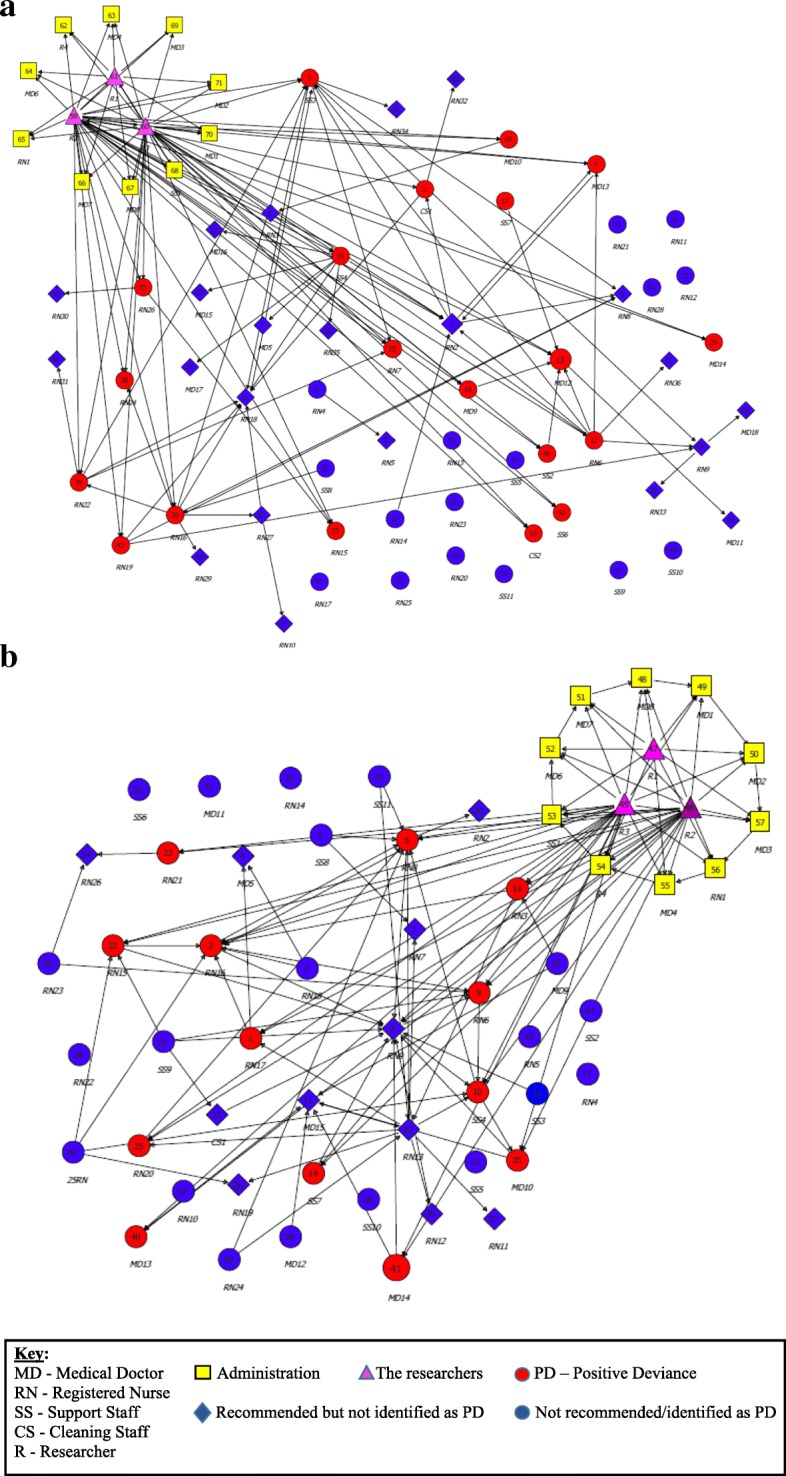
**a** The Social Network - GICU at Hadassah Medical Center-Ein Kerem Hospital. **b** The Social Network - MICU at Hadassah Medical Center-Ein Kerem Hospital. Key relevant for both social network maps. Each map has separate staff members excluding yellow and pink staff. The maps present all the participants involved in the research (management, researchers and staff from all sectors). Each arrow indicates a participant’s referral of another participant they recommended, or a participant identified by the researcher during the interviews and observations; sometimes the same team member was referred by several staff members. As shown by the maps, the research population was divided into three groups: (1) Red circles - the positive deviants (PDs) recommended by the staff and identified by the researchers; or identified only by the researchers, (2) Blue circles - staff not recommended and not identified as PDs., and (3) Blue diamonds – staff recommended but not identified as PDs

It emerged from the interviews that not everyone who was perceived by their colleagues as a PD was in fact a PD individual. A staff member who is recommended as PD by their associates but who was not identified to be PD in the study, does not necessarily work inappropriately, but may work according to the guidelines without exhibiting unique positive behaviors. In the GICU (Fig. 1a), 20 individuals were found to be PDs (ten individuals who were found to be PDs by the researchers but had not been recommended by their colleagues, additional to another ten PDs who had been recommended). In the MICU (Fig. 1b), 13 individuals were found to be PDs (four individuals were identified as PD by the researchers who had not been recommended by their colleagues, in addition to nine found to be PD who had been recommended). The PD practices identified were often specific to a unit, yet some practices could be adopted by other units.

### Gray areas on the care continuum

The interviews with representatives of the various sectors identified staff with obvious difficulties for staff, especially concerning care situations lacking clear guidelines and where a staff member interpreted or understood differently. Those issues are herewith designated “gray areas”. Fifty-five out of the 85 interviewees (65%) of interviewees from the General and Medical ICUs spoke about gray area situations. Selected quotes related the gray areas from their comments about different situations on the care continuum are presented (Additional file 2: Table S3), and the solutions proposed by the PD (Additional file 3: Table S4).

The existing Israel Ministry of Health guidelines for HH are based on the WHO’s Five Moments for Hand Hygiene, which refer to the main moments of patient care. However, there are many moments of contact on the care continuum that are not addressed by this model and where staff members were unsure how to proceed. Furthermore, because of the lack of training, specific guidelines or a clear definition of roles; staff members held different interpretations in different care situations. These conditions evoked feelings of confusion, uncertainty, and the perception of the procedures as incoherent. The gray areas on which the stakeholders focused addressed several themes as follows:

#### Lack of uniformity in infection control procedures

Some interviewees claimed the guidelines Hospital’s specific infection control unit are stricter than those of the Israeli National Center for Infection Control, and conversely sometimes the National Center’s guidelines are stricter than the hospital’s, creating a lack of uniformity and confusion among staff. For example, a senior doctor mentioned that during the procedure of inserting a central line in a patient, the guidelines sequence steps do not address all the issues that occur in practice. During that specific procedure the neck area of the patient needs to be in a sterile field, and the ultrasound (US) probe has a cable that, when moved, can enter the sterile field, thus causing contamination. The doctor suggested fixing the probe in place to prevent movement and keep the field sterile. This exemplifies the gray areas that came to light during procedures that were not covered by the guidelines (see Additional files 2 and 3: Table S3 and S4). The guidelines are also unclear and interpreted differently by staff workers regarding cleaning a patient’s room, washbasin and cleaning sinks (see Additional files 2 and 3: Table S3 and S4).

#### Vagueness in the guidelines concerning the extraction and sending of tests

Staff members repeatedly cited their confusion and uncertainty regarding obtaining, storing and submitting blood and urine samples. For instance, there is a lack of clarity about the correct way to remove samples from patients’ rooms and how to put the sample into the dedicated bag before they are sent to the laboratory; without contaminating the bag, the vacuum collection tube system (designed to send the tests) and other areas (see Additional files 2 and 3: Table S3 and S4).

#### Uncertainty as to the definitions of “clean” or “contaminated” spaces around the unit, the location of equipment after use, and responsibility for performance and lack of guidelines concerning mobile equipment

Definition of spaces within the unit was mentioned mainly in relation to the space in front of the entrance to the patient’s room. This space has a small table the staff uses for various purposes, such as: putting down disposable equipment (needles and syringes), writing instructions on monitoring pages, putting down tests and so on (see Additional files 2 and 3: Table S3 and S4). As arose from the interviews, there is confusion as to the definition of that space and the proper location of equipment and instruments within the unit after removal from the patient’s room (after use or following a malfunction). It is not clear which equipment has been sterilized and cleaned and which has not, where equipment that needs to be removed should be placed so it is clear that it has not yet been sterilized, and who is responsible (see Additional files 2 and 3: Table S3 and S4). Another point is the absence of clear guidelines and disagreement concerning the use of mobile equipment and other items within a patient’s room, such as stethoscope, cellphone, papers, stamp, etc. The staff members expressed uncertainty as to when to use, mark or disinfect these items and had different perspectives concerning the use of personal equipment in patients’ rooms (see Additional files 2 and 3: Table S3 and S4).

#### Challenges in the transition from “clean” to “dirty” areas in the course of treatment and back

This issue was mentioned only by nurses, when they described how they operate when caring for patients. All of them knew how to explain the guiding principle of their work, which is to begin by treating the patient’s “clean” areas (areas that do not involve excretions) and to end with the “dirty” areas (the digestive system), to avoid transferring bacteria from the dirty areas to the clean ones. However, the nurses described complex treatment situations that require them to move between areas: to remove gloves, sanitize hands and to continue the desired action (see Additional files 2 and 3: Table S3 and S4).

#### HH training and reminders

The issue of training was mentioned many times and in different contexts by staff members in different sectors. The physicians and nurses reported that on the one hand there are numerous training sessions, study days, and computer software teaching HH. However, they pointed to elements currently missing in the training that create confusion and deficiency. For example, the lack of customized training for each sector and for specialized work in the ICU. The interviews also raised the lack of training for staff not considered medical personnel such as cleaning staff and orderlies – who claimed that they never received systematic training regarding hygiene. The non-medical staff come in close contact with patients and their surroundings and intervening during their training process can help brake the chain of infection control (see Additional files 2 and 3: Table S3 and S4).

### PD solutions for gray areas on the care continuum

Considering the gray areas mentioned in the interviews with the different sectors, 33 individuals were identified as “positive deviants,” who, in contrast to the remaining 52 staff members who reported confusion and frustration, practiced behavioral practices that addressed the gray areas on the care continuum. A total of 14 practices that support maintenance of hygiene were discovered among the PD’s, addressing different barriers to good hygiene in the gray areas such as the use of Tegaderm sticker to immobilize and cover the tape holding the tube in place, so it doesn’t contaminate the sterile area, during insertion of a central line into the internal jugular vein (see Additional file 3: Table S4).

## Discussion

The literature is replete with attempts to design and promote customized guidelines to reduce infections during the care continuum. However, the present study suggests that despite the importance of the written guidelines, they cannot cover all the different situations that arise during the care continuum that may result in the spread of hospital infections. The lack of solutions for different areas of the care continuum creates gray areas where some staff members do not know or are unaware of what is required of them, leading to confusion, frustration, and various interpretations by them. Gurses et al. [12] found that staff compliance with unit guidelines increases when they know the accepted norms and expectations of their unit and guidelines are less vague. It is also important to clearly define roles and responsibilities for performing specific tasks and meeting guidelines [12]. Two issues that arose from the present study and were discussed by Kim et al. [33] are adapting to changing medical situations and moving between clean and contaminated areas. Most staff members stated that existing infection control guidelines do not take into account for many medical conditions they encounter daily when priorities change, such as urgent resuscitation. Staff members also pointed to the need for HH solutions in situations such as bathing the patient, when staff members’ hands go back and forth repeatedly between dirty areas (e.g. catheter) and clean areas such as the intubation tube.

Our findings indicate that written guidelines cannot be totally comprehensive as they fail to account for the dynamic nature of the work and therefore it is hard to translate them into the work environment, as guidelines cannot address all the gray areas on the care continuum. Srigley et al. [16] concurred and criticized the literature that has often focused on the planned behavior theory, whereas HH is usually an automatic, spontaneous, repetitive behavior affected by the perception of the context and environment. The movement of staff members between tasks is complex and identifying some of the specific situations when HH needs to be performed is a challenge. The study by Fuller et al. [34] tried to solve the complex continuous problems of the staff by creating hints to help the staff remember hygiene procedures. The study suggests that future interventions should be developed in cooperation with staff to build an “if then” program: “if X happens then I will do Y”.

The PD approach we used in this study addresses the need to develop practices that arise from the professional community, follows the “if then” model, and provides responses in situ to gray areas on the care continuum. The study shows it is possible to identify staff members who found solutions, big and small, that are not written or recommended in the accepted “five moments” guidelines but address “problematic” and vague situations on the care continuum. One example is taking a blood sample from a patient in an isolation room. The guidelines focus on the order of actions to be followed while performing the task, but not what happens when the sample is removed from isolation room pending transfer to the laboratory via the vacuum tube collection system located in the middle of the unit. There is a lack of uniformity as to how staff members perform this action, and each tends to interpret it differently.

In one example, we observed a nurse PD take samples from a patient’s room according to accepted procedure, then place the test tubes on a desk in the patient’s room adjacent to the exit. The nurse removed her gloves and robe, performed HH, and obtained a special plastic bag for the samples. She then placed her hand inside the bag, returned to the room entrance and picked up the samples from the inside of the bag (without direct contact between the samples and her hand or the outside of the bag) and tied it at the top. Next, she placed the samples into the vacuum tube collection system without contaminating the environment, her hands or the laboratory technician. This example displays ingenuity and effectiveness, because it demonstrates how with one small action a long cascade of infection transfer is broken and that wisdom and expertise are in the hands of community members. This solution does not require special resources, is not written in the accepted guidelines, and is a simple behavior practice that is not considered a work method that most staff members use, that grew out of conditions on the ground and can be duplicated and learned by other staff.

Our study findings indicate that there are numerous individuals who find solutions to the gray areas. The creative and practical solutions of PDs can often address barriers and difficulties on the care continuum that were raised by the staff. Because these solutions come from the community, it is very likely that people within the system will be more open to adapting them [35]. The power of a solution that comes from the community also speaks to the issue of implementing guidelines. One of the barriers that arose in the present study is healthcare workers feeling that whoever wrote the guidelines is unfamiliar with the complexity of the work on the ground. We found that identifying PD staff members has a positive effect on the enthusiasm of the staff to participate in improving infection control. Furthermore, disseminating new ideas from staff members creates an environment of eagerness to find even more constructive ideas.

It is important to note that we are not suggesting ignoring existing guidelines, on the contrary, they are the scientific building blocks that need be used in practice. Our contribution is a tool kit that can be used to minimize the existing ambiguities between the written guidelines and work practices. The tool kit is composed of PD practices demonstrated through videos, face-to-face discussions and simulations. They are recommendations taught by the PD staff to their colleagues and thus diffused throughout the work environment. The findings that rise from this study are solutions from the ground (bottom up), an important resource that can help design community-based intervention programs customized to a hospital unit profile. The PD can be used to create “unwritten guidelines” that are derived from actual people and implemented in the medical unit’s work environment. It stands to reason that this solution should be the easiest and best one to implement, since it makes sense both from a principled and practical point of view and is viewed by the staff as an efficient solution. Community involvement in building the infrastructure will lead to more openness and a multi-systemic effort to reduce infection rates in hospital units. Another study contribution is that it transfers the weight from focusing on the guidelines to focusing on practical solutions. Furthermore, as opposed to previous PD studies that focused only on the “how,” this study focuses on the “why” – the staff’s reasons and barriers; revealing the shortcomings of writing additional guidelines and structured programs and showing how the PD approach can address gaps. Previous studies focused on behaviors and less on the reasons for barriers, such as were pointed out during this research that could be resolved using the PD approach. The research contribution is also based on a variety of examples and adaptations that can be demonstrated through simulations to staff with different responsibilities in diverse units [20–23].

### Limitations

From the staff members’ interviews and observations, we found that hygiene and the prevention of HAI are considered sensitive subjects of great concern to unit staff, and even more so to hospital administrations. However, observing staff members can elicit feelings of resistance and stress and a tendency not to change behavior (the Hawthorne effect [36]). In our study we searched for the exceptional positive practices, and therefore, despite the social desire of the Hawthorne effect, it was not an issue since the staff won’t try to “hide” or change behavior because mistakes were not observed\relevant. During our observations we emphasized that the research goal was to identify the gaps and the positive behaviors and not problems with staff, thereby reducing resistance. In addition, the interviews were in hospital ICUs that have their unique features and organizational culture, and we hope the study findings can be extrapolated to other units.

## Conclusions

The present study characterized the gray areas in the care continuum that were explained by the staff, and where solutions were found through PD practices. Instead of investing in writing additional and specific guidelines for different situations and developing training programs for their implementation, it is important to encourage hospital personnel to create their own solutions for different situations on the care continuum, and to disseminate them in the units to achieve a bottom to top change.

## Additional files

Additional file 1: **Table S2.** Semi-structured interview protocol sample questions. (DOCX 17 kb)

Additional file 2: **Table S3.** Grey area themes and selected interviewees’ quotes. (DOCX 26 kb)

Additional file 3: **Table S4.** Gray area themes - PD Interviewees’ description and selected quotes. (DOCX 27 kb)

## Abbreviations

CDC: Center for Disease Control and Prevention
DAD: Discovery & action dialogue
EU: European union
GICU: General intensive care unit
HAI: Healthcare-associated infections
HH: Hand hygiene
ICU: Intensive care unit
IHI: Institute for healthcare improvement
IPC: Infection prevention and control
MICU: Medical intensive care unit
MRSA: Methicillin-resistant *Staphylococcus aureus*
PD: Positive Deviance
USA: United States of America
WASH: Water sanitation and hygiene
WHO: World health organization
