# Supplementary material for: Beyond the hospital infection control guidelines: a qualitative study using positive deviance to characterize gray areas and to achieve efficacy and clarity in the prevention of healthcare-associated infections
Source: Antimicrob Resist Infect Control. 2018 Oct 24;7:124. doi: 10.1186/s13756-018-0418-x (PMC6201509; doi:10.1186/s13756-018-0418-x)
Supplement: Supplementary file 1 — Table S2. Semi-structured interview protocol sample questions. (DOCX 17 kb) [file 13756_2018_418_MOESM1_ESM.docx]

**Table S2: Semi-structured interview protocol sample questions**

| **Number** | **Question** |
| --- | --- |
| **1** | What acute problems do hospitals deal with today? |
| **2** | Why are infections considered problematic? |
| **3** | What are your personal practices to maintain hygiene? |
| **4** | What are the challenges for the staff to maintain hygiene? (even though there are guidelines and tutorials the hand hygiene compliance rates are still low, why do you think this is the case?) |
| **5** | What do you think would help the staff cope with the challenges you mentioned? Do you have new ideas on how to maintain hygiene? |
| **6** | Are there situations during the continued care for which you found solutions to practices where no clear guidelines exist? |
| **7** | Tutorials: Who do you think should lead the tutorials? In-house staff or staff from the Infections Control Unit? |
| **8** | Could you recommend colleagues who have practices\tips\unique behaviors that help improve or maintain hygiene? |
