# Supplementary material for: Beyond the hospital infection control guidelines: a qualitative study using positive deviance to characterize gray areas and to achieve efficacy and clarity in the prevention of healthcare-associated infections
Source: Antimicrob Resist Infect Control. 2018 Oct 24;7:124. doi: 10.1186/s13756-018-0418-x (PMC6201509; doi:10.1186/s13756-018-0418-x)
Supplement: Supplementary file 2 — Table S3. Grey area themes and selected interviewees’ quotes. (DOCX 26 kb) [file 13756_2018_418_MOESM2_ESM.docx]

**Table S3: Grey area themes and selected** **interviewees' quotes**

| **Theme** | **Subtheme** | **Quotes** |
| --- | --- | --- |
| **Lack of uniformity in infection control procedures** | **The Israeli National Center for Infection Control (Ministry of Health) guidelines do not address or harmonize with all procedural steps in situ** | ***Physician****: "…There are guidelines for inserting a central line with a specific readymade kit, but there are many things that the doctors don't pay attention to, like how to place the endotracheal tube holder to prevent infection, how to move the ultrasound probe without entering the sterile field…"*  ***Physician:*** *“I don't always put on a robe. I'm not sure that practice is justified. As I understand, it is not a demand of the National Center for Infection Control. They require hand washing and gloves and only when it comes to contact isolation do you need to wear a robe. In general, there is no such requirement. I think it is not a good thing…if you require a robe in contact isolation rooms only, and for the rest of the rooms you don't wear a robe, people will notice the difference."*  ***Nurse****: “I sat in on a few meetings with the infection department…they hand down instructions and then for every tiny thing when I go into the room I need to put on a robe, it doesn't work that way… There is 100% lack of clarity (of the instructions: when to wear a robe?). Especially when it comes to the family – when do they need to wear it and when not. The whole time there is a lack of clarity."* |
|  | **Rules for cleaning patients' rooms, washbasins and sinks** | ***Nurse’s aide****: "During the whole hospitalization period, this was my problem…we need to clean the washbasin because there is soap and bacteria buildup…I clean them with the blue hand sanitizer but it doesn't help, it's meant for hands, not for stainless steel… the chlorine with the stainless steel causes a yellow stain and it doesn't clean as well… the Infection Control Unit said they would check it out but they still haven't."*  ***Nurse:*** *“The washbasins... We do the washing... So, who takes the washbasins away at the end to be sterilized? Either they stay in the rooms dirty or... I don’t know.”*    ***Nursing student: "****I don't know whether to do it or not because I don't see how other students work, but I clean the sink… sometimes they forget the urine container in the sink… no one taught me how to do it, but I think it needs to be cleaned. It’s the cleaning worker’s job…but I don't trust him, I clean the sink…"* |
| **Vagueness as to the guidelines concerning the extraction and sending of tests** | **Sending blood gas tests**.  Even though it is forbidden to leave the room and walk around the department with gloves, the nurse is required to take out the blood gas test and put it into an instrument located in the middle of the department corridor. This gives rise to the inevitable situation where she must keep her hands gloved. | ***Nurse:*** *“When you take blood gas tests... You always go out with your gloves on and the problem is that you are not supposed to... and even if I change gloves, the device is always contaminated. What you are supposed to do is inject the blood into the instrument, throw away the syringe, take off your gloves and sanitize your hands....”* |
|  | **Sending blood and urine tests**  How to exit the patient’s room with a blood/urine test prior to sending it in the container for the pneumatic tube system located in the middle of the department. There is uncertainty about the order of inserting the samples into the dedicated bag before sending them to avoid contaminating the bag, containers and other areas along the way. | ***Physician:*** *“I just saw somebody go out with a urine sample and walk around everywhere. That’s an issue I don’t know how to deal with. Maybe it should be left in the room, near the door, and at the end picked up, put in the container and sent off. Not left standing on the counter. Maybe that’s an idea. Hmm, I don’t know what doctors actually do when they move from bed to bed now.”* |
| **Uncertainty concerning equipment placement after use, responsibility for performance and guidelines for cleaning - Equipment and instruments**  The location of equipment and instruments within the department after it is taken out of the patient's room (at the end of use or following a malfunction). |  | ***Nurse:*** *“The patient is in the room, I just changed his whatever, and the saturation cable didn’t work, so I changed the saturation cable ...  so, where do I put it? (referring to the old cable she replaced) OK, the same goes for washbasins... after we wash the patients... Who takes the washbasins out at the end of washing for sterilization... they stay in the rooms and they’re dirty... I don’t know.”*    ***Physician:*** *“Handling the equipment is a big problem. The staff touches keyboards, screens, the environment... we touch the patient and then go back to the keyboard... they (the cleaning staff) clean the room well but definitely do not touch the keyboards.”*    ***Equipment technician****: "In the meantime there is no recognized sign to tell me if an appliance was cleaned or not...I'm sure there are misunderstandings and an appliance that was touched by an infected patient enters the supply room and thus became a source… (for transferring infection)"* |
| **Uncertainty about defining spaces in the department as “clean” or “contaminated”**.  This refers to confusion in how to use different spaces in the department. Especially areas in front of patients' room where there is often a small table that the staff uses for clean/not clean purposes, such as disposable equipment (needles and syringes), samples and for writing. |  | ***Nurse:*** *“Is the area outside of the patient’s room... that little table, is it clean or dirty? Because I think we treat it as a clean area, but it’s considered a dirty area... So, let’s say that’s a problematic point.”* |
| **Absence of instructions concerning mobile equipment.**  The use of mobile equipment within a patient's room (e.g. stethoscope, mobile phone, papers, signature stamp). Here too there was disagreement. The staff members expressed different positions as to the need and the urgency of using these accessories and expressed a variety of courses of action to deal with the use of personal equipment within the patient's room.  There are no general or specific guidelines concerning actions related to mobile equipment in the department. The staff knows the guidelines for hygiene after contact with the patient’s environment, but in practice, each staff member acts differently. |  | ***Physician:*** *“Our patients are also very active and need lots of things. We don't have a computer with all the patients' information in the room***^**^***, so if I want to give a medicine and I don't know, for instance, their potassium level, I need to go out.* *And the papers go in and out of the room.”*  ****** This happens in the medical ICU where there is not a complete coverage with electronic medical records, whereas in the general ICU there is complete digitalization and a computer in every room with all the information in the electronic medical record.    ***Physician:*** *“I clean the stethoscope at least once a day, but between patients if they are not in isolation I don't clean it... I admit I am not careful...  I'm not really sure what the rationale is.”*    ***Nursing aide****: "It’s 10 times more work… an isolation room is very hard. You need to enter with the paper, be careful not to touch anything when you lift the paper, clean the pen with alcohol and let it dry until you get to the supplies room, (take the supplies) and then again come back, get dressed (apron, gloves) and then again organize the supplies and enter."*  ***Nurse:*** *"…the chairs go in and out of the rooms without anyone knowing where they have been…an infected patient's chair is moved and then I find it in the staff break room, maybe the family moved it, the staff during cleaning…it contaminates the ward…"* |
| **Transition from “clean” to “dirty” areas during treatment and vice versa**  This issue was mentioned only by nurses, when they described how they operate when caring for a patient. All of them knew how to explain the guiding principle of their work, which is to begin by treating the patient's "clean" areas (areas that do not involve excretions) and to end with the "dirty" areas (the digestive system), to avoid transferring bacteria from the dirty areas to the clean areas. However, the nurses described complex care situations that require them to move between areas: to take off gloves, sanitize their hands and continue the desired action. |  | ***Nurse*:** *"Actually I try to start with the clean things, so I start with the chart and I go on to treat symptoms that don't require me to touch the patient. Then I continue on to areas that I do have to touch but not dirty things and then I work on dirty things like the catheter and stuff like that. If for example you must do suction for the patient, I try to do it first and then go on to the dirty things... and to change gloves in between the treatments. I have to say it is very difficult. With gloves it is easier to contaminate. I see it happen and I think when we take off gloves and touch the patient we feel them and know that we touched something, so I automatically clean my hands before I go somewhere else. Sometimes I also forget to do it, because I do not feel my hands are dirty. It's hard to do and I think we really have to think about it."* |
| **Training and hand hygiene**.  A need to customize training for each sector separately  (according to professional background, knowledge level and language). |  | ***Physician:*** *“I think we, the ICU staff, know the work better... and everyone who comes from the outside doesn't always know the territory and there is some resistance and less willingness to accept what they say. But, if my nursing staff tells me how to clean and why... that would make more sense to me. It's not just somebody coming in and giving us more work or giving us another procedure on the way to the patient.”*    ***Nurse:*** *“... Do training for the entire medical staff, training for each sector, according to the person's level in terms of knowledge and absorption... Take care of that... Make common guidelines between the departments, how to go in and out of patients’ rooms.”*    ***Nurse’s aide****: "An outside contractor gives the nurses aides' training. This morning I talked to him and told him that when he instructs a worker he should do it properly because people aren’t getting proper training."*  ***Nursing aide****: "There is no training for this kind of thing. We never got special training about isolation rooms, they just told us to put on an apron and gloves and discard them when we leave the room."*    ***Cleaner:*** *"…If the worker is from Hadassah, it's their responsibility… If we are contract workers, they will cause us lots of problems. If you come tomorrow there will be a new boss, they change all the bosses..."* |
