# Supplementary material for: Beyond the hospital infection control guidelines: a qualitative study using positive deviance to characterize gray areas and to achieve efficacy and clarity in the prevention of healthcare-associated infections
Source: Antimicrob Resist Infect Control. 2018 Oct 24;7:124. doi: 10.1186/s13756-018-0418-x (PMC6201509; doi:10.1186/s13756-018-0418-x)
Supplement: Supplementary file 3 — Table S4. Gray area themes - PD Interviewees’ description and selected quotes. (DOCX 27 kb) [file 13756_2018_418_MOESM3_ESM.docx]

**Table S4: Gray area themes - PD Interviewees' description and selected quotes**

| **Theme** | **Subtheme** | **Description of the PD (*= video documented)** | **Quotes** |
| --- | --- | --- | --- |
| **Lack of uniformity in infection control procedures** | **The Israeli National Center for Infection Control (Ministry of Health) guidelines do not address or harmonize with all procedural steps in situ** | *Guidelines for inserting a central line do not address many of the procedural steps needed to maintain strict aseptic technique during the insertion process. A senior doctor gave ideas that were video documented to help reduce infections during the insertion process:   1. A sterile field is created in the patient's neck area during insertion of the central line into the internal jugular vein. The problem is that the tape holding the tube in place usually reaches the neck area and enters the sterile field, thus potentially contaminating the line during insertion. The doctor suggested to use a Tegaderm sticker to immobilize and cover the tape, so it doesn't contaminate the sterile area. 2. The second suggestion uses the Ultrasound (US) probe to guide central line insertion. The US probe has a cable connecting it to the screen, which during the procedure is covered with a sterile plastic sheath. The problem is that when the US probe is moved back and forth during the procedure, the cable and sheath move in and out of the sterile field, thus increasing the risk for contaminating the sterile field. The doctor's solution was to translocate the probe within the field when not in use in a way that does not move the cable in and out. |  |
|  | **Rules for cleaning patients' rooms, washbasins and sinks** | **The cleaning worker** cleans the chairs in the breakroom with a septic pad before the staff sits down. This task was not in her guidelines, but she explained that many people sit on the chairs and it is not nice to sit without it being clean. | ***Nurse’s aide****: "I put a chlorine tab in the sink every night … so it doesn't have any bacteria. When the cleaning staff cleans after a patient leaves… there will be little contamination and not a lot…"*  ***Nurse’s aide****: "…every morning I clean the rooms with chlorine, the carts, it's something that I love, not everyone does it, but I really love it….in every room I clean the supplies cart, the bed and the surrounding area. I did it before they asked because it also affects the nurse's mood when she enters the patient’s room and sees everything is clean and ordered."* |
| **Vagueness as to the guidelines concerning the extraction and sending of tests** | **Sending blood gas tests**.  Even though it is forbidden to leave the room and walk around the department with gloves, the nurse is required to take out the blood gas test and put it into an instrument located in the middle of the department corridor. This gives rise to the inevitable situation where she must keep her hands gloved. | **Nurse:** After performing the patient’s blood gas test, you should leave the room holding the syringe in a gloved hand. When you get to the blood gas machine you should inject the blood directly into it using one gloved hand; then remove one glove, and do HH on that hand, then scan the patient's ID using barcode instrument while waiting for the test results. After receiving the results, you should throw the syringe into the designated bin using the gloved hand, remove the glove and do HH. This prevents contaminating the instrument and surrounding surface.    *This solution concerns sending blood gas tests with a blood gas machine located in the middle of the department (documented by video) |  |
|  | **Sending blood and urine tests.**  How to exit the patient’s room with a blood/urine test prior to sending it in the container for the pneumatic tube system located in the middle of the department. There is uncertainty about the order of inserting the samples into the dedicated bag before sending them to avoid contaminating the bag, containers and other areas along the way. | **Nurse:** After taking a patient’s blood sample in their room, the nurse lays the sample on the counter adjacent to the room, takes off her robe and gloves, and performs HH. She then leaves the room to get a dedicated bag for sending the samples.  In order to prevent contaminating the outside of the bag with the samples, she turns the bag inside out keeping her hand wrapped in the bag, picks up the samples from the inside of the bag, and with the other hand closes the bag - i.e. using the bag as if it was a glove and then de-gloving it over the sample tubes so that it turns inside out.  *This solution prevents contamination of the sample when transferred from the patient’s room via the pneumatic system to the laboratory. |  |
| **Uncertainty concerning equipment placement after use, responsibility for performance and guidelines for cleaning - Equipment and instruments**  The location of equipment and instruments within the department after it is taken out of the patient's room (at the end of use or following a malfunction). |  |  | ***Equipment technician****: "A sticker is a good idea, for example a green circle sticker that you put on the appliance and then you know that someone cleaned the appliance…"* |
| **Uncertainty about defining spaces in the department as “clean” or “contaminated”**.  This refers to confusion in how to use different spaces in the department. Especially areas in front of patients' room where there is often a small table that the staff uses for clean/not clean purposes, such as disposable equipment (needles and syringes), samples and for writing. |  | **Nursing aide:** He suggested a solution, transferring supplies from an outside cart into the room cart, while keeping one hand “clean” for the outside cart and the gloved “contaminated” hand handles things inside the room, so as not to contaminate the clean supplies. | ***Nursing aide:*** *"Let's say we’re filling up the room cart with equipment, every morning we do a round with the main cart to bring supplies to the rooms. I check what is missing, bring the room cart to the door, open the drawer, take off my gloves, use hand sanitizer and let my hands dry. Then I wear a glove on the hand that only touches the room cart using my other hand to move equipment from the main clean cart (outside the room) … after I finish I go over the main cart with a cloth…"* |
| **Absence of instructions concerning mobile equipment.**  The use of mobile equipment within a patient's room (e.g. stethoscope, mobile phone, papers, signature stamp). Here too there was disagreement. The staff members expressed different positions as to the need and the urgency of using these items and expressed a variety of courses of action to deal with the use of personal equipment within the patient's room.  There are no general or specific guidelines concerning actions related to mobile equipment in the department. The staff knows the guidelines for hygiene after contact with the patient’s environment, but in practice, each staff member acts differently. |  | **The quotes in the following column apply to the following details:**  Keeping a pen and flashlight in the room  Marking chairs  Placing a chlorine-soaked sheet at the entrance to the room  Paperwork  Stethoscope  **Nurse:** She suggested keeping a marker and a flashlight permanently in the room to prevent infections caused by bringing equipment in and out*.*    **Nurse:** A nurse suggested a simple and practical solution – to mark chairs that belong in patients' rooms to prevent their transfer to other places without prior disinfection. | **Nurse:***“I like it very much when there is a pen and flashlight in the room, I don't like to take them out of my pocket even if I'm treating a noninfectious patient.”*  ***Nurse****: "When we have a new admission and you don't yet have stickers because the patient isn’t in the system, then you prepare stickers ahead of time to separate nose and feces samples... and you attach the stickers on the swabs... I don't have to touch and write on the swabs when I'm clean”*  ***Nurse:*** *“We had a very serious CPE patient who came from another city, so I thought about it, with people going in and out so many times... to put chlorine on the floor. I thought about the X-ray machine going in and out of the room and its wheels are not cleaned... So, let's put a rag with chlorine at the entrance and then the wheels will go over it and get cleaned as they step over the chlorine soaked rag.”*    ***Nurse:*** *“If I know in advance in the morning that I’m going to work with somebody infected then I prepare the chart and I put my stamp on it in advance and then I don't need to take my signature stamp into an infected room. The chart is out… and I stamp it in advance and put my stamp back. And then I go back to the room with the chart and the meds to dispense them.”*    ***Nurse:*** *“First I go into the patient's room and observe how they are lying, what they are getting. When I finish all those things, if the patient is not in isolation, I write myself a note of the meds they are getting because we are not computerized. If the patient is in isolation, I take a picture of the chart with my phone. I prepare everything the patient needs and enter to give it."*    ***Physician:*** *“Stethoscopes are the biggest problem I think but with clostridium what I actually learned from one of our senior physicians is to put on a glove… So, in isolation rooms and in general I put a glove over the stethoscope and listen through the glove. Then I disinfect before and after…  I wash my hands and then the stethoscope right away and I don't have to especially open something and look for it.”*    ***Physiotherapist****: "I disinfect. I adapted a work method that in units with infected patients I disinfect everything! From the earbud down the whole pipe (of the stethoscope), I always carry alcohol pads in my pocket and that way it is easier to access…"* |
| **Transition from “clean” to “dirty” areas during treatment and vice versa**  This issue was mentioned only by nurses, when they described how they operate when caring for a patient. All of them knew how to explain the guiding principle of their work, which is to begin by treating the patient's "clean" areas (areas that do not involve excretions) and to end with the "dirty" areas (the digestive system), to avoid transferring bacteria from the dirty areas to the clean areas. However, the nurses described complex care situations that require them to move between areas: to take off gloves, sanitize their hands and continue the desired action. |  | *Three nurses from the department identified a solution for this problem, installing disinfectant on the headboard instead of or in addition to the one near the sink. This solution does not yet occur in the department, but it does not require special resources. According to the nurses, this would increase the use of disinfectants and they would not have to leave the patient in the middle of treatment to perform HH. | ***Nurse:*** *“In the hospital room there is only one alcohol antiseptic hand rub next to the sink, which means that if I'm next to a patient I have to go to the sink and perform HH and then come back....and then while I’m doing something I have to go to the sink and back. So, if the disinfectants were closer, even at the foot of the bed... Because if I’m very busy, especially if I’m in the middle of washing or treating a wound, then I can't leave the patient and go disinfect my hands...”* |
| **Training and hand hygiene**.  A need to customize training for each sector separately  (according to professional background, knowledge level and language). |  |  | ***Nurse:*** *“Learn from the best. We are used to seeing what people do wrong, when they make mistakes. It raises resistance. For instance, if a department does something unusual or new, and it works, we can try to transfer it to other places. We can learn from each other.”*    ***Nurse:*** *"I think the secret is ongoing assimilation, and giving as many workshops as possible, even simulations. I think it will help at some point because talking about theory is very nice but the more we deal with the practice, it can become a routine and integrated work process.”* |
